# A Novel Method for ECG-Free Heart Sound Segmentation in Patients with Severe Aortic Valve Disease

**DOI:** 10.3390/s25113360

**Published:** 2025-05-27

**Authors:** Elza Abdessater, Paniz Balali, Jimmy Pawlowski, Jérémy Rabineau, Cyril Tordeur, Vitalie Faoro, Philippe van de Borne, Amin Hossein

**Affiliations:** 1 Laboratory of Physics and Physiology, Department of Cardiology, Hôpital Erasme, Hôpital Universitaire de Bruxelles, 1070 Brussels, Belgium; 2 Laboratory of Image Synthesis and Analysis (LISA), Université libre de Bruxelles, 1050 Brussels, Belgium; 3 Research Unit in Cardio-Respiratory Physiology, Exercise & Nutrition, Faculty of Human Movement Sciences, Université libre de Bruxelles, 1070 Brussels, Belgium; 4 Department of Kinesiology and Health Sciences, University of Waterloo, Waterloo, ON N2L 3G1, Canada

**Keywords:** aortic stenosis, aortic regurgitation, automatic detection, phonocardiography, telemedicine, valvular heart disease

## Abstract

Severe aortic valve diseases (AVD) cause changes in heart sounds, making phonocardiogram (PCG) analyses challenging. This study presents a novel method for segmenting heart sounds without relying on an electrocardiogram (ECG), specifically targeting patients with severe AVD. Our algorithm enhances traditional Hidden Semi-Markov Models by incorporating signal envelope calculations and statistical tests to improve the detection of the first and second heart sounds (S1 and S2). We evaluated the method on the PhysioNet/CinC 2016 Challenge dataset and a newly acquired AVD-specific dataset. The method was tested on a total of 27,400 cardiac cycles. The proposed approach outperformed the existing methods, achieving a higher sensitivity and positive predictive value for S2, especially in the presence of severe heart murmurs. Notably, in patients with severe aortic stenosis, our proposed ECG-free method improved S2 sensitivity from 41% to 70%.

## 1. Introduction

Calcific aortic valve disease (AVD) is a life-threatening condition with an increasing incidence worldwide [1]. It is defined by echocardiographic evidence of stenosis or regurgitation due to a degenerative calcification of the aortic valve [1]. This condition is often undiagnosed in asymptomatic patients [2]. However, the timely diagnosis and treatment of AVD is crucial as a late presentation is associated with a worse prognosis due to ventricular remodeling and hemodynamic consequences [2]. The lower mortality observed in countries with a high socio-demographic index, a measure of societal development, illustrates the benefit of better access to healthcare [3].

Traditional semiology, based on a physical examination and cardiac auscultation with a stethoscope, may be useful for AVD screening. However, it relies on the proficiency of care providers and may not allow a timely diagnosis [4,5]. Moreover, current imaging techniques used in clinical practice are not suitable nor cost-effective for large-scale screening: the quality of echocardiography depends on the operator’s expertise and the echogenicity of the patient, the computed tomography (CT) scan is an irradiating technique, and cardiac magnetic resonance imaging (MRI) is a time-consuming, expensive, and less available technique [6]. In addition, none of these techniques are accessible to people living far away from a healthcare institution.

Phonocardiography (PCG) is a portable technology that may enhance the screening of these pathologies [7,8]. It enables the detailed recording and analysis of heart sounds produced by the blood flow during the opening and closing of heart valves. It allows the extraction of heart sound information that reveals valvular diseases, such as the timing of heart valve closure, the frequency content of heart sounds, and the presence of diastolic or systolic murmurs [9].

The fundamental heart sounds (FHSs) are called S1 and S2. The first is initiated by the closure of the mitral and tricuspid valves at the beginning of the systole, occurring immediately after the R-peak of the electrocardiogram (ECG) [10]. The second is initiated by the closure of the aortic and pulmonary valves at the beginning of the diastole, occurring approximately at the end-T-wave of the ECG [10]. S3 and S4 may be observed as additional heart sounds following S2, indicating increased ventricular filling pressure or decreased left ventricular compliance, respectively [11]. In a healthy population, the majority of the frequency content of the FHS is in the range of 20–200 Hz [12]. However, in a population presenting murmurs, the range is 15–700 Hz [12]. The correct segmentation of FHS can be complex due to murmurs, clicks, FHS replication, and S3 and S4 sounds, which have similarities in morphology and the frequency ranges [10]. Acquiring heart sounds is also susceptible to various types of interferences, notably friction between the equipment and human skin, along with random noises such as breath, lung, and bowel sounds [10].

The segmentation of the FHS is a critical step in the automated analysis of PCG. Various automatic segmentation methods for PCG signals have been described in the literature and tested on different datasets with several types of murmurs, making a direct comparison difficult. Springer et al. proposed a probabilistic approach by using a Hidden Semi-Markov Model (HSMM) and by extracting multiple signal features [13]. They evaluated the performance of their algorithm on a dataset including subjects with normal valves and subjects with mitral valve prolapse, achieving an F1-score of 95.63%, which is the harmonic mean of the positive predictive value (PPV) and sensitivity (Se). Building upon this foundation, Shukla et al. used an HSMM with different features based on segmenting the signal in impulses and increased the F1-score by 3.37% on the same dataset [14]. Jain et al. instead used a Tunable Quality Wavelet Transform to decompose the signal and prioritize FHS, yielding satisfactory results on a dataset of subjects with valvular heart diseases (VHDs) [15]. However, this was achieved on only 134 cardiac cycles from subjects with aortic stenosis or regurgitation; indeed, many were eliminated before segmentation. Xu et al. then presented a method based on the signal envelope calculation using K-means clustering and a dynamic threshold with which to identify S1 and S2 peaks. Nevertheless, they noted that their algorithm could not achieve good results on abnormal heart sounds with murmurs of high amplitude [16]. Finally, Arjoune et al. developed a threshold-based segmentation method using the Shannon Energy envelogram [17]. On a large dataset of children with and without murmurs of different types, an average Se of 97.44% and 97.69% was achieved for detecting S1 and S2 sounds, respectively. Several studies also presented machine-learning methods; however, their effectiveness significantly depended on the availability of a large dataset [18].

Despite promising results, there are limited data on the automatic segmentation of heart sounds in an older population with a high frequency and amplitude of murmurs. This paper attempts to address the challenge of accurately segmenting the FHS in noisy real-world recordings from patients with severe AVD, characterized by high-intensity murmurs and, in some severe aortic stenosis, a soft or absent S2 due to reduced leaflet mobility [19]. To achieve this, a novel segmentation method is presented, incorporating an HSMM followed by an envelope calculation to improve the detections of FHS. The proposed segmentation approach is evaluated on one of the largest published datasets and on a newly acquired dataset. It is then compared with the current state-of-the-art segmentation algorithms.

## 2. Materials and Methods

### 2.1. Algorithm Description

#### 2.1.1. Overview of the Proposed Method

**Figure 1:**
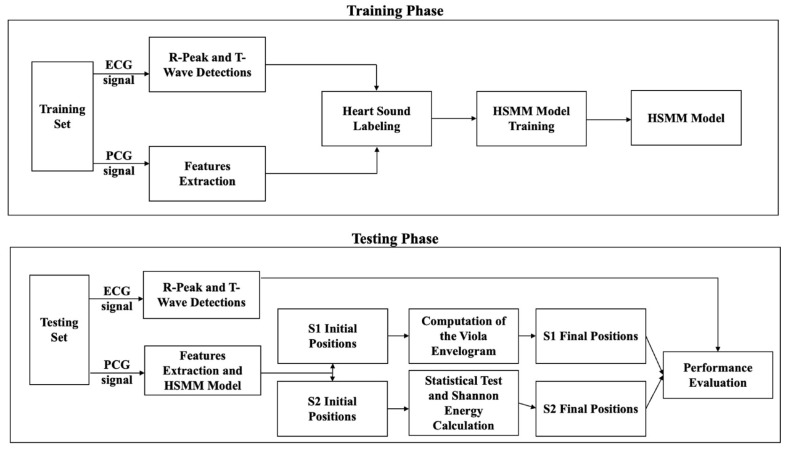
Workflow of the proposed method for ECG-free heart sound segmentation, divided into two main phases: training and testing. In the training phase, ECG and PCG signals from a training dataset undergo R-peak and T-wave detections (ECG), as well as feature extraction (PCG). These features are used for heart sound labeling, which facilitates the training of a Hidden Semi-Markov Model (HSMM). The trained HSMM model is then applied in the testing phase, where ECG is only used for performance evaluation. Initial positions of the first and second fundamental heart sounds (S1 and S2) are identified, followed by wavelet envelope analysis and Shannon energy calculation to refine these positions. The final S1 and S2 positions are determined and evaluated for segmentation performance.

The method integrates an HSMM with post-processing techniques based on signal envelope analysis and statistical refinement. The segmentation pipeline consists of a training phase, during which the HSMM is fitted using ECG-annotated PCG signals, and a testing phase, in which only PCG signals are used for segmentation [13,14]. Refinement steps are then applied to enhance the detection accuracy of S1 and S2, which is particularly useful in the presence of murmurs and attenuated valve sounds. The overall workflow is illustrated in Figure 1.

All processing operations described in this study were performed using MATLAB R2018b (MathWorks, Inc., Natick, MA, USA).

#### 2.1.2. Pre-Processing

PCG signals were pre-processed using a third-order Chebyshev band-pass filter between 40 and 450 Hz to remove noise and retain relevant cardiac components. The filtered signals were normalized by dividing by their maximum absolute amplitude to minimize inter-individual variability. For ECG signals—used exclusively during training and evaluation—we applied a second-order Butterworth filter between 0.5 and 100 Hz, followed by notch filtering to eliminate powerline interference and harmonics. R-peaks were detected using the Pan and Tompkins algorithm [20], implemented via the BioSigKit MATLAB toolbox [21], while the ends of T-waves were identified using a template-matching technique based on an ensemble-averaged ECG beat [22,23].

#### 2.1.3. Initial Segmentation Using HSMM

Initial segmentation of heart sounds was performed using an extended four-state HSMM, modeling the cardiac cycle as a sequence of S1, systole, S2, and diastole. This model incorporates features derived from the Hilbert envelope of the PCG signal, which highlights the energy bursts associated with FHS [13]. To further enhance the identification of heart sounds, we computed the kurtosis of the Hilbert envelope using a 0.2-s sliding window with 1 ms overlap, as proposed by Shukla et al. [14]. A zero-frequency filter was applied to isolate impulsive features corresponding to heart sounds. The characteristics of the filter have already been described in [14].

The HSMM was trained on five five-minute PCG+ECG recordings from five different individuals presenting various cardiac pathologies. No recording appeared in both the training and test sets. During training, ECG-based annotations were used to label S1 (occurring shortly after the R-peak) and S2 (around the end of the T-wave), allowing the HSMM to learn the transition probabilities and duration distributions associated with each cardiac phase, as described by [13].

#### 2.1.4. Proposed Method to Improve Detections of First Heart Sound Positions

To enhance S1 detection in the presence of systolic murmurs, an additional envelope analysis was applied.

The signal envelope is calculated using the Viola integral, initially used for the fast detections of images [24], and, now, heart sound analysis [16,25]. The Viola integral is particularly useful in this context as it provides a smoothed cumulative representation of signal energy over time, enabling more robust identification of localized peaks. Its cumulative nature helps suppress high-frequency noise and transient artifacts, which is especially valuable in pathological recordings where systolic murmurs can mask or distort the actual S1 peaks. In this respect, a physiologically meaningful temporal window (*L_T_*) over which the cumulative envelope is computed is needed; therefore, *L_T_* should approximate the duration of S1. Based on the empirical observations reported in [16], the typical duration of the S1 sound was estimated, leading to the formulation of Equation (1), where Fs is the PCG sampling frequency and the factor 0.5 reflects the proportional scaling derived from their findings.

(1) $$L_{T}=0.5\times 0.1\times Fs$$

Next, the mean sequence $\bar{X_{T}}(m)$ of the signal $X_{T}(k)$ is expressed in Equation (2):

(2) $$\bar{X_{T}}(m)=\frac{1}{2L_{T}+1}\sum_{k=m-L_{T}}^{m+L_{T}}X_{T}\left(k\right)$$

Then, the Viola integral envelope is given by Equation (3):

(3) $$E_{T}\left(m\right)=\frac{1}{2L_{T}+1}\sum_{k=m-L_{T}}^{m+L_{T}}{\left(X_{T}\left(k\right)-\bar{X_{T}}(m)\right)}^{2}$$

with *m* = $L_{T}$, $L_{T}+1$, …, ${M-1-L}_{T}$, where *M* is the signal length.

The sequence was then normalized to produce the final envelope in Equation (4):

(4) $$E\left(m\right)=\frac{E_{T}\left(m\right)}{max(\left|E_{T}\left(m\right)\right|)}$$

The next step aims to highlight the peaks of the Viola integral envelope (*E*(*m*)) signal using a first-order average Shannon energy (*ASE*). *ASE* has been widely used for extracting the heart sound envelope in prior studies [17,26,27,28].

Shannon energy emphasizes the medium-amplitude components more effectively as compared to the high-amplitude components. It also attenuates the low-amplitude components [8]. Thus, the Shannon-energy-based envelope method helps to identify the fundamental heart sound with medium amplitude, especially in pathological cases, where one of the FHS may have a lower amplitude than the other one. The first-order Shannon energy is given by Equation (5):

(5) $$E\left(t\right)=-X\left(t\right)\;\ln(X(t))$$

In Equation (5), *X*(*t*) is empirically defined as *X*(*t*) = 0.4 *E*(*m*) to avoid a double-peak problem at the location of S1 sound, as described by [16].

To eliminate small spikes, Shannon energy is converted into the normalized average Shannon energy (*ASE*) [16]. To do so, the average Shannon energy (${Es}_{\left(t\right)}$) is computed with a window length (*N*) set to 20 ms, with an overlap of 10 ms, as in Equation (6):

(6) $${Es}_{\left(t\right)}=\frac{1}{N}\sum_{j=i-N}^{i+N}E\left(j\right)\ln(E(j))$$

with *E(j)* being the Shannon energy of the Viola integral envelope. After normalization, we obtain the *ASE* in Equation (7):

(7) $$ASE(t)=\frac{{Es}_{\left(t\right)}-mean({Es}_{\left(t\right)})}{std({Es}_{\left(t\right)})}$$

*Es_(t)_* is the average Shannon energy, *mean*(${Es}_{i}$) is the average of the calculated ${Es}_{i}$, and *std*(${Es}_{i}$) is its standard deviation.

For each initially detected S1 interval by the HSMM segmentation, we first compute the local maximum of the *ASE* within the S1 interval. This maximum corresponds to the most probable acoustic peak of S1. The algorithm then searches backward from this peak to identify the last sample where the envelope falls below 70% of the peak value. This heuristic is based on the observation that, in pathological signals, S1 can exhibit a gradual buildup rather than a sharp onset. By anchoring the new start time to a fraction of the peak height, the method adapts to varying morphologies of S1. Let *t_max_* denote the index of the maximum of *ASE* within the S1 segment. Then, the new S1 start index is computed as presented in Equation (8):

(8) $$t_{start}^{\prime }=t_{max}-∆t,\;\mathrm{where}\;ASE\left(t_{max}-∆t\right)\leq 0.7*ASE(t_{max})$$

If no such index exists, the original boundary is retained. The end of S1 is symmetrically shifted to preserve the original duration.

#### 2.1.5. Proposed Method to Improve Detections of Second Heart Sound Positions

In individuals with VHD, a murmur is usually present. For example, murmurs due to aortic valve stenosis (AS) occur during systole, and murmurs due to aortic valve regurgitation (AR) occur during diastole. Therefore, using the algorithm described so far, part of the murmur may be mistakenly identified as S2 during segmentation. To address this, the following method was implemented. Based on the initial HSMM segmentation of the FHS, for each recording, we calculated the average duration $S_{12}$, defined as the time interval between S1 and S2. To determine whether this $S_{12}$ duration follows a Gaussian distribution, we computed the distribution parameters (mean and standard deviation) from the entire dataset. As explained in [29], the duration $S_{12}$ follows a Gaussian distribution depending on the heart rate and, more specifically, on the duration of systole. Once the Gaussian distribution is established, we evaluated whether the initial $S_{12}$ duration fell within the 95% confidence interval of the established Gaussian distribution. If it did not, this indicated that the $S_{12}$ duration for the new recording deviates from the expected pattern, prompting further analysis.

In that case, the second-order *ASE* was calculated over a sliding window length (*N*) set to 20 ms with 50% overlap between successive windows. Let *X* denote the normalized band-pass filtered PCG. *E′_i_* is computed as follows:

(9) $${E^{\prime }}_{i}=-\frac{1}{N}\sum_{i=1}^{N}{X(i)}^{2}log({X\left(i\right)}^{2})$$

Then, *ASE* is computed as follows:

(10) $$ASE={E^{\prime }}_{i}-mean({E^{\prime }}_{i})$$

Then, *ASE* was normalized, and denoted NASE, by dividing it by the maximum value within *ASE*. For each heartbeat detected with abnormal $S_{12}$, let ${t_{1}}^{\prime }$ be the time corresponding to the start of the S2 interval and ${t_{2}}^{\prime }$ the time corresponding to a point located at the center of the diastolic interval. Within the window [${t_{1}}^{\prime }$; ${t_{2}}^{\prime }$], the algorithm identifies local NASE energy lobes by detecting changes in the sign of the derivative (zero-crossings), which are interpreted as candidate components of the S2 sound.

If at least two candidate lobes are found, the algorithm selects the final lobe pair as the most likely representation of the true S2. This decision is based on the hypothesis that late diastolic energy lobes often better represent S2 in patients with delayed valve closure due to stenosis.

### 2.2. Datasets

#### 2.2.1. PhysioNet/Computing in Cardiology (PhysioNet/CinC) Challenge 2016 Dataset

The proposed segmentation approach was first evaluated on the PhysioNet/CinC Challenge 2016 dataset [10]. It includes 409 PCG recordings with automatically annotated R-peak and T-wave labels from ECG waveforms, based on the agreement between four different automatic detectors, as described in [13]. The PCG and ECG signals are resampled at 1000 Hz. Only signals lasting more than 2 s are saved. The heart sounds were recorded at various locations on the chest of 121 subjects, including recordings from healthy subjects with (n = 118) or without (n = 117) benign murmur, as well as VHD patients with mitral valve prolapse (n = 134), AVD (n = 17), and other pathological conditions (n = 23). The age and gender of the subjects are unknown.

#### 2.2.2. ARTIK Dataset

The proposed segmentation approach was then evaluated on a newly acquired dataset called “ARTIK”, as part of a prospective study currently being conducted in accordance with the declaration of Helsinki and approved by the Ethics Committee of Hôpital Erasme-ULB (P2023/346/B4062023000197) and is registered on ClinicalTrials.org identifier NCT06286358. This dataset includes subjects recruited between November 2023 and August 2024. Healthy adults without known VHD nor murmur at cardiac auscultation were recruited. Adult subjects with severe AVD were recruited during their consultation or hospitalization at Erasme University Hospital in Brussels, mostly before a surgical or transcatheter aortic valve replacement. Atrial fibrillation and frequent extrasystoles were considered as exclusion criteria. All subjects gave their written informed consent for inclusion before they participated in the study. All AVD diagnoses were first made by the clinical cardiology team of the hospital and then verified by one experienced sonographer of the research team using a Vivid E95 (GE Healthcare, Milwaukee, WI, USA). Aortic valve area and gradient were measured by simplified continuity and Bernoulli equations, respectively. Left ventricular ejection fraction was assessed by 2D biplane Simpson’s or visual estimation when image quality did not allow Simpson’s. Valvular regurgitation was graded according to the guidelines of the European Society of Cardiology, using the proximal isovelocity surface area (PISA) method, when it was reliable [30]. Mixed AVD, defined as the coexistence of at least moderate AS and at least moderate AR, were, respectively, assigned to AS or AR group according to the predominant anomaly. Recordings of five-minute synchronized PCG and ECG were collected immediately after the echocardiography as a result of an AD Instrument PL3508 PowerLab 8/35 (Bella Vista, New South Wales, Australia) with one input from an MLT201 Cardio Microphone and a second input from ECG electrodes (DII derivation). All recordings were obtained by placing the Cardio Microphone on the aortic area, centered at the second right intercostal space, guided by stethoscope auscultation. For each healthy person, a single recording was made in supine position, while, for each patient with heart valve disease, two recordings were made: one in supine position and one in sitting position. For this work, only the supine position was analyzed. PCG and ECG recordings were saved in text format at a sampling rate of 1000 Hz.

### 2.3. Re-Implementation of Other State-of-the-Art Methods

To benchmark performance, we re-implemented four segmentation methods from previous publications [14,15,16,17], and their performances have been evaluated on the ARTIK dataset. Methods were selected based on recency, citation count, and robustness across various heart sound types. Methods that performed well across different types of heart sounds and datasets were prioritized to ensure generalizability. We used a weighted scoring system based on these factors to identify methods that demonstrated the best balance of performance and applicability to our dataset.

### 2.4. Performance Metrics

As described in previous publications, we used the synchronous ECG to evaluate the performance of the heart sound segmentation algorithms [13,14]. The reference positions for S1 and S2 sounds were based on R-peak and end of T-wave positions. If the start of an S1 detected by the algorithm was within 100 ms after the corresponding R peak in the ECG, it was counted as a true positive (TP). Similarly, if the center of an S2 detected by the program was within an interval of 100 ms around the corresponding end of T-wave, it was counted as a TP. False negatives (FN) were defined as the number of S1 or S2 sounds that were not detected. False positives (FP) were defined as the number of detected S1 and S2 sounds that did not happen in the windows defined around the R-peak and the T-wave, respectively.

The Se and the PPV were then computed as follows:

(11) $$Se=\frac{TP}{TP+FN}\times 100$$

(12) $$PPV=\frac{TP}{TP+FP}\times 100$$

The overall performance of the algorithm was measured in terms of F1-score (see Equation (11)). The F1-score represents the harmonic mean of PPV and Se. It takes into account both FP and FN and is particularly useful when one of the two classes is unbalanced.

(13) $$F1score=2\frac{PPV\times Se}{PPV+Se}$$

## 3. Results

### 3.1. PhysioNet/CinC Challenge 2016 Dataset

Table 1 and Table 2 present a summary of the results obtained on the PhysioNet/CinC dataset in the identification of S1 and S2 heart sounds, respectively. Each table details the number of TP, FP, and FN, as well as Se, PPV, and F1-score, based on the algorithm used for each group of subjects.

**Figure 2:**
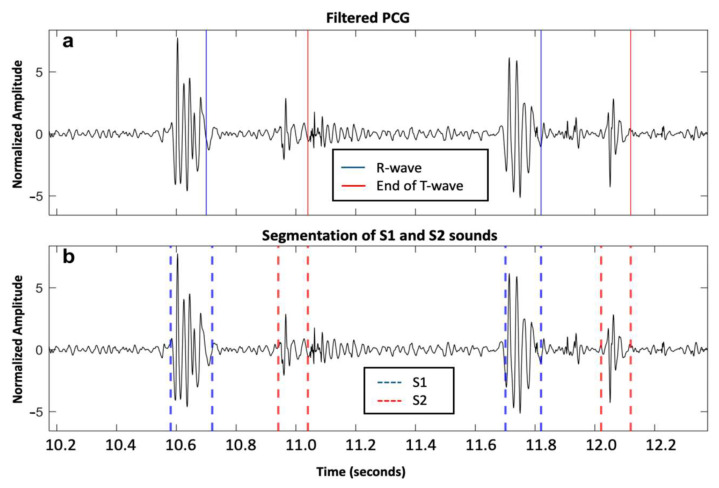
Two cardiac cycles on a phonocardiography (PCG) signal recorded from a healthy individual from the PhysioNet/CinC Challenge 2016 dataset: (**a**) Filtered PCG signal with R-wave and end of T-wave reported as blue and red solid lines, respectively; and (**b**) segmentation of first and second fundamental heart sounds (S1 and S2) reported as blue and red dashed lines, respectively, based on the proposed algorithm.

**Figure 3:**
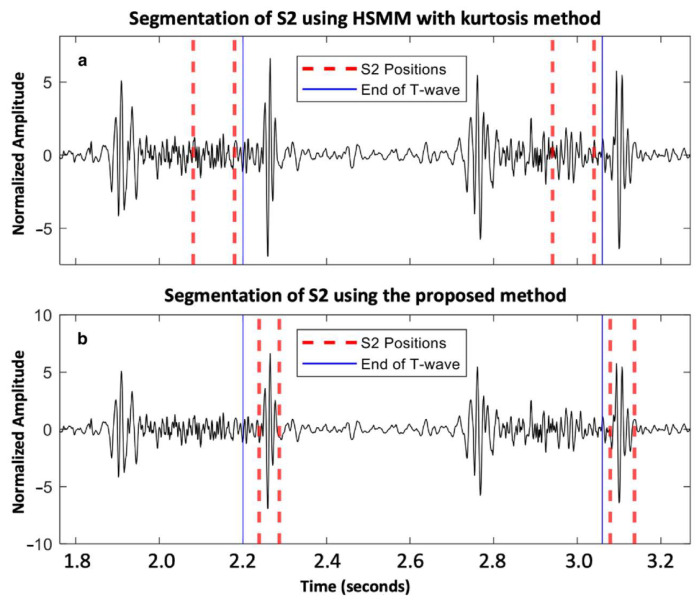
Aortic valve disease subject from the PhysioNet/CinC Challenge 2016 dataset: (**a**) segmentation of S2 using HSMM with kurtosis method [14]; and (**b**) segmentation of S2 using the proposed method.

A total of 11,469 S1 and 11,313 S2 were analyzed. Our proposed method for S1 and S2 detections reached F1-scores of 95.9% and 93.9%, respectively. For the healthy group, we achieved an Se of 96.16% and PPV of 96.40% for S1 detection, while we achieved an Se of 94.87% and PPV of 95.26% for S2 detection. For the VHD group, the performance was slightly lower, with an Se and PPV of 94.06% and 96.21%, respectively, for S1 detection, and 91.31% and 92.73%, respectively, for S2 detection. An example of the segmentation of two cardiac cycles in a healthy subject is shown in Figure 2. An example of S2 segmentation in a subject with AVD is shown in Figure 3.

### 3.2. ARTIK Dataset

#### 3.2.1. Description of the ARTIK Dataset

A total of 44 adults were included: 9 were healthy subjects assigned to the control group and 35 had AVD, including 8 isolated AR (1 severe, 6 moderate, and 1 mild), 21 isolated AS (20 severe and 1 moderate), and 6 mixed AVD (5 predominant severe AS and 1 predominant severe AR). The subjects’ characteristics, including sex, age, body height, body weight, BMI, and echocardiographic findings, are described in Table 3.

#### 3.2.2. Performances of the Algorithms on the ARTIK Dataset

Table 4 and Table 5 summarize the results obtained in the recognition of S1 and S2 sounds, respectively. A distinction was made between the populations of healthy individuals, those with AS, and those with AR. Specifically, each table outlines the number of TP, FP, FN, Se, PPV, and F1-score according to the group of individuals studied. On the ARTIK dataset, a total of 15,931 first and 15,302 s FHS were analyzed.

For healthy individuals, the proposed method achieved an Se, PPV, and F1-score of 98.09%, 97.84%, and 97.96%, respectively, for S1 recognition, as well as 96.68%, 95.16%, and 95.91%, respectively, for S2 recognition.

In subjects with AR, the proposed method achieved an overall Se, PPV, and F1-score of 95.29%, 94.94%, and 95.29%, respectively, for S1 recognition, as well as 92.44%, 90.23%, and 91.32%, respectively, for S2 recognition.

However, in people with AS, the algorithm’s performance was lower. The approach achieved an Se, PPV, and F1-score of 82.47%, 83.48%, and 82.97%, respectively, for S1 recognition, as well as 70.27%, 70.43%, and 70.35%, respectively, for S2 recognition. It still showed a notable improvement when compared to the baseline HSMM with kurtosis method in subjects with AR and AS. Especially in the latter group, the performance of S2 detection improved by 29.28%, 29.44%, and 29.36% for Se, PPV, and F1-score, respectively.

**Figure 4:**
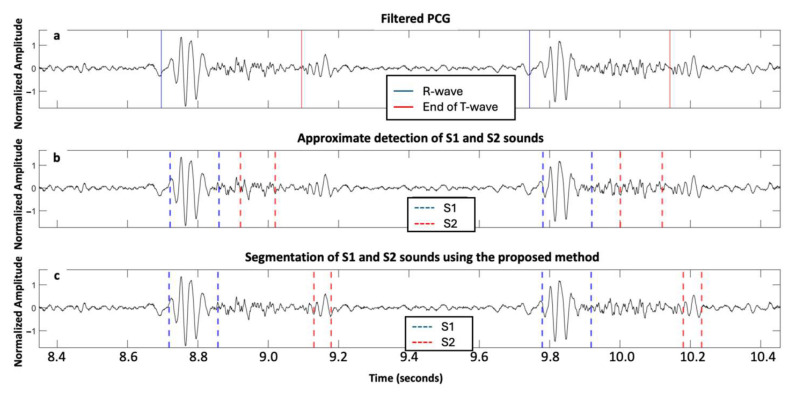
Signals acquired from one subject with severe aortic stenosis, mild aortic regurgitation, and mild mitral regurgitation from the ARTIK dataset: (**a**) filtered PCG with R-peak and end of T-wave annotated; (**b**) segmentation of first and second fundamental heart sounds (S1 and S2) based on the HSMM with kurtosis method; and (**c**) segmentation of S1 and S2 using the proposed approach.

**Figure 5:**
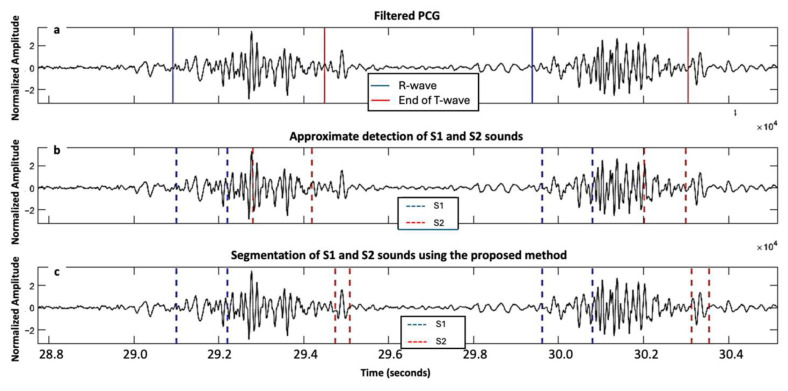
Signals acquired from a second subject with severe aortic stenosis, mild aortic regurgitation and mild mitral regurgitation from the ARTIK dataset: (**a**) filtered PCG with R-peak and end of T-wave annotated; (**b**) segmentation of first and second fundamental heart sounds (S1 and S2) based on the HSMM with kurtosis method; and (**c**) segmentation of S1 and S2 using the proposed approach.

Figure 4 and Figure 5 illustrate the segmentation of two cardiac cycles in PCG signals from two subjects with severe AS.

## 4. Discussion

This study’s main contribution is the development of an advanced segmentation algorithm that extends the HSMM with kurtosis framework. This algorithm is followed by an envelope calculation and statistical testing to improve the detections of FHS found by the HSMM with kurtosis.

The algorithm was tested on one of the largest available datasets and a new dataset collected specifically for this study, ensuring a broad performance assessment. The results showed that the proposed method provides reliable results, particularly in challenging cases involving noise and atypical heart sound patterns. Indeed, in these cases, the proposed method notably improved the detection of S2 heart sounds while maintaining the detection reliability of S1 heart sounds.

### 4.1. Performance for S1 and S2 Detections on the PhysioNet/CinC Challenge 2016 Dataset

On 11,469 cardiac cycles from the PhysioNet/CinC Challenge 2016 dataset, our proposed method for S1 and S2 detections reached an F1-score of 95.9% and 93.9%, respectively. Our approach builds on the HSMM with kurtosis method described by Shukla et al. [14], which is based on Springer’s method [13]. While our results are consistent with those reported by Springer et al., they fall slightly short of the performance reported by Shukla et al. However, it is important to note that Shukla’s results were derived from only 534 cardiac cycles, representing just 4% of the dataset. Similarly, Xu et al. [16] reported higher performance, but their testing was limited to only 1000 cardiac cycles. Overall, our method did not demonstrate significant improvements over existing algorithms for S1 and S2 detections on this dataset, as the baseline performance is already notably high in the literature.

### 4.2. Performance for S1 and S2 Detections on the ARTIK Dataset

On the ARTIK dataset, the performance of our proposed method for S1 detections is equivalent to the baseline HSMM with kurtosis method in each group. Further comparisons with the methods [15,16,17] we re-implemented in the scope of this study suggest that HSMM-based approaches are better suited for S1 detections in AVD (Table 4). The results for S1 detections are very satisfactory in severe AR, with both an Se and PPV of around 95%. However, the precision of the detections is lower in severe AS, with both an Se and PPV of around 83% for S1 detections. One explanation may be the prominent diamond-shaped systolic murmur that may mimic FHS in severe AS subjects, reducing the Se and PPV for S1 detections.

Our proposed method showed an identical performance to the HSMM with kurtosis method for S2 detection in healthy subjects, with an Se of 96.68%, a PPV of 95.16%, and an F1-score of 95.91% (Table 5). However, it is well-known that severe AS is often characterized by a reduced or absent S2 due to the reduced mobility of the leaflets, making the detection of S2 particularly challenging. The true prevalence of this feature has not been extensively studied. In a series of 397 subjects with AS, mainly composed of severe diseases, nearly 10% had an absent S2 [31]. Our method demonstrated an improvement by nearly 30% of Se and PPV for S2 detections in subjects with severe AS, compared to the HSMM with kurtosis method. This improvement can be attributed to the signal envelope refinement steps and dynamic threshold adjustments. The results for S2 detections remained lower than expected in severe AS, with an Se of around 70%. However, it is consistent with previous studies, highlighting the difficulty of detecting S2 in the presence of strong systolic murmurs or when S2 is undetectable or severely attenuated in severe AS [17,32]. Further comparisons with the methods we re-implemented in the scope of this study [15,16,17] suggest that our approach is still better suited for S2 detections in severe AVD (Table 5). It is noteworthy that the method based on the Shannon energy envelope outperformed other previous methods in this specific case, revealing that the segmentation process benefits from the envelope analysis.

**Figure 6:**
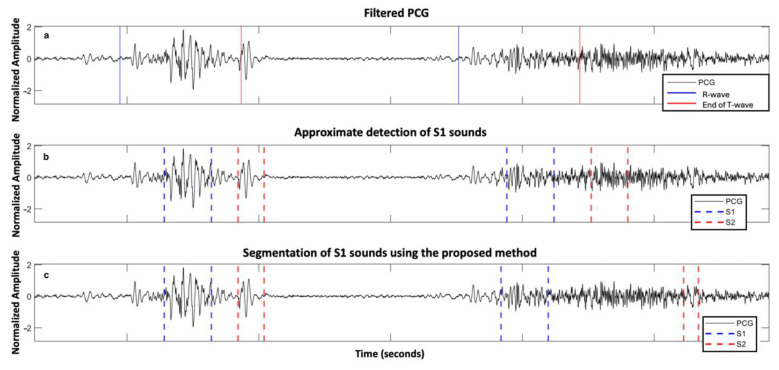
Example of errors in segmenting two heartbeats in a subject with mixed aortic valve disease with predominant severe aortic stenosis from the ARTIK dataset: (**a**) PCG filtered with R-peaks and end of T-waves reported; (**b**) approximate segmentation of S1 and S2 sound; and (**c**) segmentation of S1 and S2 sound using the proposed approach.

The supervised nature of the algorithm implies that the model performance depends strongly on the diversity of the training data. In our dataset, only six patients exhibited mixed AVD (five attributed to the AS group and one to the AR group, according to the predominant anomaly), restricting our ability to draw strong conclusions regarding performance in these cases. Mixed and multiple valvular heart diseases, in which systolic and diastolic murmurs may coexist and overlap with the FHS, remain particularly challenging. These scenarios highlight the need for pathology-specific adaptations such as frequency-domain filtering or dynamic thresholding to enhance segmentation robustness. Figure 6 illustrates this difficulty, showing segmentation errors across two heartbeats in a patient with mixed AVD. During the first beat, only a systolic murmur is present, while the second beat includes both systolic and diastolic murmurs, complicating the identification of S1 and S2.

### 4.3. Comparison to Other Methods in the Literature

Arjoune et al. developed a threshold-based segmentation method with the Shannon energy envelogram [17]. They tested their algorithm on the CirCor dataset [33] by developing a method to remove noisy segments from the recordings. The dataset consisted of 5272 heart sound recordings from four primary auscultation sites in 1568 participants aged 0 to 21 years (mean ± SD: 6.1 ± 4.3 years). On that dataset, the noise-robust algorithm demonstrated an overall Se of 97.22% when evaluated on recordings with murmurs, compared to 97.69% for recordings without murmurs. However, on the ARTIK dataset, this re-implemented method performed worse in each group, particularly in the AS and AR groups (Table 4 and Table 5). We also observed that, despite normalizing the signals to reduce the inter-patient variability, the threshold used for lobe identification in the original article was not the optimal parameter. To maximize performance, we adjusted the thresholds, which varied between 0 and 0.25 depending on the patient, making it difficult to automate the process.

In another study, Jain et al. employed a Tunable Quality Wavelet Transform (TQWT) to decompose the signal into twenty levels [15]. A specific level was then chosen to prioritize FHS over murmurs. This algorithm was evaluated on a dataset that included various types of VHD, such as mitral, aortic, and tricuspid valve diseases, and produced satisfactory results. However, the selection of the decomposition level depends on the specific type of disease. While this method effectively emphasizes FHS when the appropriate decomposition level is selected, the results on the ARTIK dataset were highly variable due to the fixed decomposition level (Table 4 and Table 5). Therefore, to achieve optimal outcomes, the TQWT decomposition level should be tailored to each patient’s specific parameters.

Finally, Xu et al. [16] identified the S1 and S2 peaks using a dynamic threshold and K-mean clustering, achieving an F1-score of 98.54% for S1 and 97.97% for S2 in healthy subjects. In people with VHD, the respective F1-scores for S1 and S2 were 99.10% and 98.48%. To obtain these results, thirty different PCG signals were randomly selected from the PhysioNet/CinC Challenge 2016 dataset previously described. Unfortunately, we have not been able to reproduce their results in the ARTIK dataset (Table 4 and Table 5). Whilst in healthy individuals, the F1-score was still around 88%, in the case of AR or AS, the presence of murmurs blurred the distances between the peaks found for S1 and S2, used for K-means clustering, making the method susceptible to failure. We also noticed that the method had a lower performance in case of high heart rate variations during the record, with the envelope failing to distinguish clear peaks at locations of the FHS.

The performance of all these algorithms on the ARTIK dataset demonstrates a noticeable drop when confronted with more complex cases of advanced VHD, compared to healthier cases. This highlights the importance of testing algorithms on signals from real-world clinical cases, where patients can present with severe or less typical conditions. Ensuring that these algorithms are validated against diverse and challenging clinical data is crucial for their robustness and applicability in everyday medical practice.

Although we prioritized transparent and interpretable signal processing techniques, we acknowledge the growing potential of compact deep-learning models (e.g., CNNs) for heart sound segmentation. In recent years, the latter approach has made advancements in the automatic detections of heart sounds, specifically S1 and S2. Convolutional neural networks (CNNs) and recurrent neural networks (RNNs) are among the most widely used architectures in this domain. In a study by Renna et al., a CNN-based model achieved an Se of 93.9% for S1 detections and 94.0% for S2 detections on the PhysioNet/CinC 2016 dataset [34]. Most of the publications in the field focus on the classification of normal/abnormal heart sounds rather than their segmentation [35]. The main advantage of these models is their ability to automatically learn features from raw PCG data, reducing the need for extensive manual preprocessing. However, deep-learning models typically require large annotated datasets, which are not always available, especially for pathological heart sounds. Despite their performance, a key limitation of deep-learning-based algorithms is their susceptibility to overfitting, especially when dealing with small or imbalanced datasets. Additionally, their complexity makes them computationally expensive, limiting their applicability in low-resource settings. Nevertheless, the ongoing integration of deep-learning methods into clinical tools could enhance the accuracy and scalability of heart sound analysis in the future. Accordingly, future studies may include direct comparisons with deep-learning approaches to better contextualize the benefits and trade-offs of our proposed method.

### 4.4. Limitations and Perspective

Despite the promising performance of our ECG-free heart sound segmentation method, several limitations should be acknowledged.

The HSMM model was trained on a small cohort (five subjects), which may limit its generalizability. The diversity of the input data might have been insufficient to cover all possible variations in heart sound signals. Nonetheless, its ability to maintain a consistent performance across two independent datasets is encouraging. Future work will involve training and validating the model on a substantially larger and more diverse cohort to address inter-patient variability in heart sounds, particularly with varying severity and types of VHD, and with mixed and multiple VHDs. One potential improvement could involve the use of k-fold cross-validation to maximize algorithm generalization across different subsets of the dataset.

**Figure 7:**
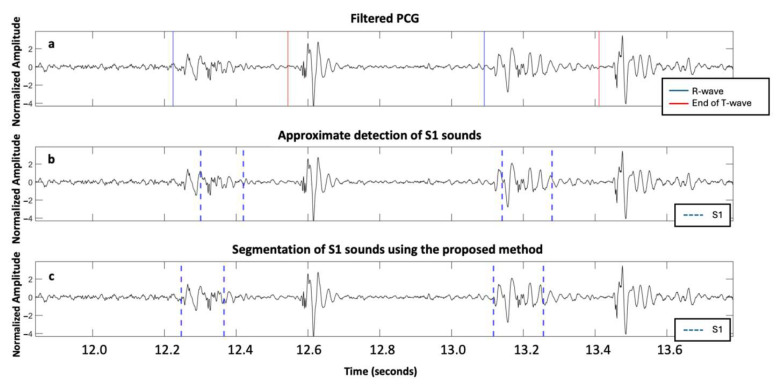
Mild aortic regurgitation patient from the ARTIK dataset: (**a**) filtered PCG with R-peaks and end of T-waves reported; (**b**) approximate segmentation of S1; and (**c**) accurate segmentation of S1 using the proposed approach.

Additionally, ECG was used as a reference for defining true S1 and S2 to assess the performance of the different methods, though it is not a perfect gold standard. We recognize that relying on ECG to approximate S1 and S2 timing may introduce inaccuracies due to variable electromechanical delays, particularly in patients with severe AVD. A fixed threshold of 100 ms after the R-peak (for S1) or around the end of T-wave (for S2) was used to enable comparability with previous studies [13,14]. However, this threshold may lack the precision required to reliably distinguish actual heart sounds from adjacent noise or murmur components. This limitation can lead to either an overestimation or an underestimation of the algorithm’s performance. For example, in Figure 7, S1 is segmented over two successive cardiac cycles of a patient with mild aortic regurgitation. In both the baseline HSMM with kurtosis method [14] and our proposed approach, the S1 sound was detected within 100 ms of the corresponding R-peak, and, thus, classified as a TP, despite evident differences in timing and morphology. Consequently, S1 is classified as a TP in all instances, even though the actual detections are different. Future work should explore performance under multiple threshold conditions (e.g., ±50 ms and ±75 ms) and examine which threshold best reflects clinically meaningful accuracy. A more precise annotation of heart sounds, ideally based on expert consensus and echocardiography-based timing of valve closure events synchronized with PCG recordings, could help establish a more accurate gold standard for validation. Nonetheless, even expert annotations are subject to uncertainty, particularly when murmur masking or a poor signal quality are present.

In our work, inaccuracies in R-peak and T-wave end detection could affect both HSMM training and evaluation. However, we applied a uniform annotation process across all methods to ensure consistency and fairness. Additionally, for the training set, all ECG-based annotations were visually inspected and confirmed.

While the current implementation is suitable for validation purposes, it has not been optimized for computational speed. Processing a 5-min recording requires approximately 4.3 min on a standard workstation (Intel i7, 16 GB RAM). Further optimization will be required for deployment in real-time or portable applications.

The automatic segmentation of the FHS is a critical first step in PCG analysis. The development of an ECG-free heart sound segmentation method has the potential to broaden the accessibility and effectiveness of cardiovascular screening, particularly in low-resource settings. First, by increasing the accuracy of S1 and S2 detections, this method could be integrated into traditional stethoscope use, potentially enhancing the diagnostic capabilities of frontline healthcare providers. Indeed, the more accurate boundary detection of FHS may facilitate the identification of murmurs occurring between or outside these boundaries. Additional work is needed to develop a computerized method for associating specific murmur patterns with a particular VHD. Second, reliable S2 detection enables its characterization. For example, a diminished, delayed, or absent S2 may suggest the presence of a severe AS [19]. As such, the development of an effective PCG-based first-line screening tool may support earlier clinical suspicion and referral for confirmatory imaging, such as echocardiography, CT, or cardiac MRI. This is especially valuable in settings where access to advanced diagnostic tools is limited, enabling timely triage and potentially improving patient outcomes.

Further work should perform additional real-world testing, especially in severe VHD, to validate the robustness of this method in clinical settings. Heart rate fragmentation (HRF), characterized by excessive beat-to-beat variability, may be present in older patients with severe AVD and comorbidities [36]. Such variability could interfere with the HSMM’s duration modeling and potentially disrupt state transitions. Future work should evaluate whether HRF affects segmentation performance and whether adaptations are needed to maintain robustness in fragmented rhythms.

## 5. Conclusions

In this article, we present a new ECG-free segmentation method incorporating an HSMM followed by an envelope calculation to improve the detections of FHS. On the PhysioNet/CinC 2016 dataset, which contained a minority of AVDs of unknown severity, our proposed algorithm was equivalent to other methods presented in the literature. However, on the predominantly severe AVD subjects from the newly acquired “ARTIK” dataset, the proposed algorithm showed a better Se and PPV for S2 segmentation than other methods we re-implemented. By improving the accuracy of FHS segmentation, the proposed method can potentially improve the diagnosis and monitoring of patients with AVD, ultimately contributing to better patient outcomes.

## Figures and Tables

**Table 1:** Results of S1 sound detections in the PhysioNet/CinC Challenge 2016 dataset with the newly proposed method. The numbers of True Positives (TP), False Negatives (FN), False Positives (FP), Sensitivity (Se), Positive Predictive Value (PPV), and F1-score are reported for the healthy and the Valvular Heart Disease (VHD) groups.

Group	TP	FP	FN	Se	PPV	F1-Score
Healthy group	6879	257	275	96.16%	96.40%	96.28%
VHD group	4059	160	256	94.06%	96.21%	95.13%

**Table 2:** Results of S2 sounds detections in the PhysioNet/CinC Challenge 2016 dataset with the newly proposed method. The numbers of True Positives (TP), False Negatives (FN), and False Positives (FP), Sensitivity (Se), Positive Predictive Value (PPV), and F1-score are reported for the healthy and the Valvular Heart Disease (VHD) groups.

Group	TP	FP	FN	Se	PPV	F1-Score
Healthy group	6715	334	363	94.87%	95.26%	95.07%
VHD group	3867	303	368	91.31%	92.73%	92.02%

**Table 3:** Description of the ARTIK dataset’s population (n = 44). Values are expressed as number of subjects or as medians and interquartile ranges [Q1, Q3], for control, aortic stenosis (AS), and aortic regurgitation (AR) groups. Echocardiographic data are reported: the maximum velocity of the flow through the aortic valve (AV Vmax), the mean pressure gradient through aortic valve, the AV area, the vena contracta of aortic regurgitation (AR), the regurgitant volume, the presence of associated mitral regurgitation (MR), and the presence of altered ejection fraction (EF). BMI: body mass index.

Characteristics	Control Group	AS Group	AR Group
Number of subjects	9	26	9
Men (%)	44	69	44
Age (years)	28 [23, 30]	75 [69, 79]	64 [53, 60]
Body height (cm)	170 [166, 179]	165 [161, 169]	168 [163, 176]
Body weight (kg)	65 [60, 80]	78 [69, 89]	70 [53, 77]
BMI (kg/m^2^)	22 [21, 25]	28 [25, 31]	23 [21, 24]
AV Vmax (m/s)	-	4.2 [4.0, 4.6]	1.8 [1.6, 2.2]
AV Mean Gradient (mmHg)	-	43 [39, 50]	6 [5, 14]
AV Area (cm^2^)	-	0.8 [0.6, 0.9]	2.5 [2.1, 2.7]
AR Vena Contracta (mm)	-	-	5 [3, 5]
AR Regurgitant Volume (mL)	-	-	46 [40, 70] ^a^
Number of associated mild MR	-	16	4
Number of associated moderate MR	-	3	0
Number of subjects with mild reduced EF (41–49%)	-	2	0
Number of subjects with reduced EF (≤40%)	-	2	0

^a^ AR regurgitant volume could be measured using PISA method in only 5 subjects with AR.

**Table 4:** S1 sound detections using different methods on the ARTIK dataset. The results are given for each of the methods that we re-implemented: HSMM with kurtosis method, Shannon method, Tunable Quality Wavelet Transform (TQWT) method, K-means method, and our newly proposed method. The numbers of True Positives (TP), False Negatives (FN), False Positives (FP), Sensitivity (Se), Positive Predictive Value (PPV), and F1-score are reported for the healthy, the Aortic Regurgitation (AR), and the Aortic Stenosis (AS) groups.

Group	Algorithm	TP	FP	FN	Se	PPV	F1-Score
Healthy group	HSMM with kurtosis [14]	2305	52	46	98.04%	97.79%	97.92%
	Shannon [17]	2106	520	245	89.10%	79.60%	84.21%
	TQWT [15]	1587	260	764	67.50%	85.92%	75.61%
	K-means [16]	2058	88	293	87.54%	95.90%	91.53%
	Proposed method	2306	51	45	98.09%	97.84%	97.96%
AR group	HSMM with kurtosis [14]	4172	288	250	94.35%	93.54%	93.94%
	Shannon [17]	2522	1366	1901	57.10%	64.90%	60.80%
	TQWT [15]	2466	849	1957	55.75%	74.39%	63.74%
	K-means [16]	3261	641	1162	73.73%	83.57%	78.34%
	Proposed method	4235	226	188	95.29%	94.94%	95.29%
AS group	HSMM with kurtosis [14]	9215	1916	2052	81.79%	82.79%	82.28%
	Shannon [17]	5415	3119	5852	48.06%	63.45%	54.69%
	TQWT [15]	4056	2748	7211	36.00%	59.61%	44.89%
	K-means [16]	5671	3266	5596	50.33%	63.46%	56.14%
	Proposed method	9292	1839	1975	82.47%	83.48%	82.97%

**Table 5:** S2 sound detections using different methods on the ARTIK dataset. The results are given for each of the methods that we re-implemented: HSMM with kurtosis method, Shannon method, Tunable Quality Wavelet Transform (TQWT) method, K-means method and the newly proposed method. The numbers of True Positives (TP), False Negatives (FN), False Positives (FP), Sensitivity (Se), Positive Predictive Value (PPV), and F1-score are reported for the healthy, the Aortic Regurgitation (AR), and the Aortic Stenosis (AS) groups.

Group	Algorithm	TP	FP	FN	Se	PPV	F1-Score
Healthy group	HSMM with kurtosis [14]	2240	114	77	96.68%	95.16%	95.91%
	Shannon [17]	3626	546	275	88.30%	79.19%	83.49%
	TQWT [15]	1423	852	894	61.42%	62.55%	61.98%
	K-means [16]	2062	309	255	88.99%	86.97%	87.97%
	Proposed method	2240	114	77	96.68%	95.16%	95.91%
AR group	HSMM with kurtosis [14]	3712	516	415	89.94%	87.80%	88.86%
	Shannon [17]	3626	1140	501	87.86%	76.08%	81.55%
	TQWT [15]	2672	1060	1455	64.74%	71.60%	68.00%
	K-means [16]	2861	671	1266	69.32%	81.00%	74.71%
	Proposed method	3815	413	312	92.44%	90.23%	91.32%
AS group	HSMM with kurtosis [14]	4517	6502	6503	40.99%	40.99%	40.99%
	Shannon [17]	7705	8881	3315	69.92%	46.45%	55.82%
	TQWT [15]	3612	4945	7408	32.78%	42.21%	36.90%
	K-means [16]	5166	4136	5854	46.88%	55.54%	50.84%
	Proposed method	7744	3252	3276	70.27%	70.43%	70.35%

## Data Availability

The datasets used and analyzed during the current study are available from the corresponding author upon reasonable request.

## Abbreviations

The following abbreviations are used in this manuscript:

AR	Aortic valve regurgitation
AS	Aortic valve stenosis
ASE	Average Shannon energy
AVD	Aortic valve disease
CT	Computed tomography
ECG	Electrocardiogram
FHS	Fundamental Heart Sounds
FN	False negatives
FP	False positives
HSMM	Hidden Semi-Markov Model
TN	True negatives
TP	True positives
MRI	Magnetic resonance imaging
NASE	Normalized average Shannon energy
PCG	Phonocardiogram, phonocardiography
PPV	Positive predictive value
Se	Sensitivity
